# Pandemic A/H1N1v influenza 2009 in hospitalized children: a multicenter Belgian survey

**DOI:** 10.1186/1471-2334-11-313

**Published:** 2011-11-07

**Authors:** Sophie Blumental, Elisabeth Huisman, Marie-Coralie Cornet, Christine Ferreiro, Iris De Schutter, Marijke Reynders, Ingrid Wybo, Benoît Kabamba-Mukadi, Ruth Armano, Dominique Hermans, Marie-Cécile Nassogne, Bhavna Mahadeb, Christine Fonteyne, Gerlant Van Berlaer, Jack Levy, Didier Moulin, Anne Vergison, Anne Malfroot, Philippe Lepage

**Affiliations:** 1 Pediatric Infectious Diseases Unit, Hôpital Universitaire des Enfants Reine Fabiola (HUDERF), Brussels, Belgium; 2 Pediatric Pneumology and Infectious Diseases Department, Universitair Ziekenhuis Brussel (UZB), Brussels, Belgium; 3 Department of Pediatrics, Cliniques Universitaires St-Luc (UCLouvain), Brussels, Belgium; 4 Department of Pediatrics, Hôpital Universitaire St-Pierre, Brussels, Belgium; 5 Microbiology Unit, Hôpital Universitaire St-Pierre, Brussels, Belgium; 6 Microbiology Unit, UZB, Brussels, Belgium; 7 Microbiology Unit, UCLouvain, Brussels, Belgium; 8 Department of Pediatrics, HUDERF, Brussels, Belgium; 9 Intensive Care Unit, HUDERF, Brussels, Belgium; 10 Emergency and Intensive Care Unit, UZB, Brussels, Belgium; 11 Infection Control Unit, HUDERF, Brussels, Belgium

## Abstract

**Background:**

During the 2009 influenza A/H1N1v pandemic, children were identified as a specific "at risk" group. We conducted a multicentric study to describe pattern of influenza A/H1N1v infection among hospitalized children in Brussels, Belgium.

**Methods:**

From July 1, 2009, to January 31, 2010, we collected epidemiological and clinical data of all proven (positive H1N1v PCR) and probable (positive influenza A antigen or culture) pediatric cases of influenza A/H1N1v infections, hospitalized in four tertiary centers.

**Results:**

During the epidemic period, an excess of 18% of pediatric outpatients and emergency department visits was registered. 215 children were hospitalized with proven/probable influenza A/H1N1v infection. Median age was 31 months. 47% had ≥ 1 comorbid conditions. Febrile respiratory illness was the most common presentation. 36% presented with initial gastrointestinal symptoms and 10% with neurological manifestations. 34% had pneumonia. Only 24% of the patients received oseltamivir but 57% received antibiotics. 10% of children were admitted to PICU, seven of whom with ARDS. Case fatality-rate was 5/215 (2%), concerning only children suffering from chronic neurological disorders. Children over 2 years of age showed a higher propensity to be admitted to PICU (16% vs 1%, p = 0.002) and a higher mortality rate (4% vs 0%, p = 0.06). Infants less than 3 months old showed a milder course of infection, with few respiratory and neurological complications.

**Conclusion:**

Although influenza A/H1N1v infections were generally self-limited, pediatric burden of disease was significant. Compared to other countries experiencing different health care systems, our Belgian cohort was younger and received less frequently antiviral therapy; disease course and mortality were however similar.

## Background

On March 2009, in Mexico, a novel recombinant influenza strain (A/H1N1v) of swine origin was discovered as an infective agent in humans [1]. This new virus spread rapidly, first to USA and Canada, then all over the world, causing the "new 2009 influenza A/H1N1v pandemic" [2]. Worldwide, the burden of disease was significant and subsequent efforts from health care systems were required to face an overload of patient's consultations as well as to implement vaccination and surveillance programs. Although consequences of the pandemic were less dramatic than initially feared, the World Health Organization (WHO) estimated that the virus was responsible of at least 17700 deaths worldwide and the Centers for Disease Control and Prevention (CDC) reported 59 millions infected people in the USA [3-5]).

During this A/H1N1v flu wave, children and young adults were identified as a particular risk group. They presented a higher attack rate than older adults [6] and a greater mortality rate than previously observed with classical seasonal flu [7,8]. Several reports on influenza A/H1N1v in pediatric settings have now been published [9-12], but information on clinical presentation and severity of infection in European children remains limited [13-15]. However, these data could be of the great interest to guide future recommendations for vaccination and antiviral therapy during forecoming flu seasons.

Belgium experienced the influenza A/H1N1v epidemic from July 2009 to January 2010. Pandemic vaccine (adjuvanted Pandemrix^®^) was only available after the peak occurred in October and was given with priority to risk groups (defined as health care workers, pregnant women and chronically ill patients) [16]. According to our national surveillance system, around 214531 people were infected, 733000 could benefit from vaccination and 19 deaths were attributable to the virus [17].

In this context, we conducted a multicenter study analyzing influenza A/H1N1v pediatric cases hospitalized in four tertiary medical centers of Brussels, Belgium. Our study aimed to offer a comprehensive description of influenza A/H1N1v infection in children, in the light of other recently published data from countries experiencing different health care systems [9-12].

## Methods

### Study design and population

In collaboration with infection control units and microbiology laboratories, we prospectively registered all proven and probable pediatric cases of influenza A/H1N1v infections hospitalized in four tertiary facilities of Brussels (Hôpital Universitaire des Enfants Reine Fabiola, Universitair Ziekenhuis of Brussels, Cliniques Universitaires Saint-Luc and Hôpital Saint-Pierre). These facilities totalize 406 pediatric beds, representing 80**% **of the total pediatric beds available in Brussels (about 1 million inhabitants in 2009). Moreover, three of the hospitals have a Pediatric Intensive Care Unit (PICU) where critically-ill children from other hospitals of Brussels and the surrounding areas are referred to (in total 32 PICU beds available).

The study period extended from July 1, 2009, to January 31, 2010. Children were included if they were aged from 0 to 18 years, presented with clinical symptoms compatible with influenza (fever and/or respiratory signs/symptoms) and had either positive PCR results for influenza A/H1N1v (proven cases), or an antigen and/or a positive culture for influenza A (probable cases). The latter cases were included because virtually no other seasonal influenza A viruses were circulating in Belgium during the epidemic period (less than 0.4%, data from the Belgian National Institute of Public Health). Moreover, specific H1N1v PCR confirmation was no longer carried out routinely at the end of the epidemic, due to the high cost of this testing and the limited number of cases after December 2009.

### Data collection and definitions

Data were collected retrospectively from patients' medical files using a standardized questionnaire.

A pre-existing co-morbidity was defined as a chronic condition requiring long term medication or medical follow up. Co-morbidities were listed based upon CDC H1N1 flu guidelines http://www.CDC.gov/h1n1flu.htm and other recent publications [8]. Co-morbidities were not mutually exclusive, so that a child could participate in several categories.

Fever was defined as a central temperature above 38° Celsius.

Nosocomial infection was defined as a proven or probable case occurring after more than 48 hours of hospitalization.

### Microbiological testing

Respiratory samples collection included nasopharyngeal aspirates, nasopharyngeal flocked swabs (Copan Diagnostics, Corona, CA), throat flocked swabs (Copan Diagnostics, Corona, CA) and sputum.

Antigen testing was assessed by immunochromatographic rapid antigen detection (RAT) in three of the four centers or also by direct immunofluorescence (DIF) technique (Argene SA, Verniolle, France) in one of them. Both DIF and immunochromatography use highly sensitive monoclonal antibodies directed against either influenza A or B nucleoprotein antigens [18]. RAT was performed using two different testing: the Coris Influ-A&B Respi-Strip (Coris Bioconcept, Gembloux, Belgium) and the Binax Now influenza A & B (Binax Inc., Inverness medical, Maine, USA). Direct antigen testing was unavailable in the fourth participating hospital, representing 26% of our cohort of patients.

Viral culture was performed on the three following cell lines: Vero, MRC-5 and LLC-MK2 in two centers; and MDCK, Hep-2, MRC-5 and LLC-MK2 in a third hospital, as described elsewhere [19]. In the fourth center which used DIF and RAT for antigen detection, respiratory samples were not cultured as antigen detection was followed directly by real-time PCR. (This center represented 22% of the cohort).

Biomolecular testing consisted, for three of the four centers, firstly in detection of influenza A virus by a home-made real-time RT-PCR (RT-PCR InflA) targeting the matrixprotein-coding gene and consequently by specific detection of the circulating pandemic variant using two monoplex real-time RT-PCR assays as described in the Centers of Disease Control (CDC) protocol: the SW InfA PCR (SWINE) and the SW H1 PCR *(RT-PCR A/H1N1) *[20]. In the fourth center (26% of samples), a commercial available PCR kit was used directly for detecting the pandemic strain: "Swine influenza virus (sw H1N1) Real-time PCR" (Diagenode Diagnostics, Liège, Belgium).

### Statistical analysis

Statistical analyses were performed using Graph Pad Prism Software, Inc, 2003, San Diego, USA. Chi square or Fisher's exact tests were used to compare non continuous variables and Mann Whitney u- test was used to compare continuous variables. A two-tailed p-value less than 0.05 was considered as statistically significant.

### Ethical Consideration

Approval of the Medical Ethics Committees of the four hospitals was obtained before starting the study. A code number was attributed to each child so that data collected remained strictly confidential.

## Results

### Epidemiological Data

**Figure 1:**
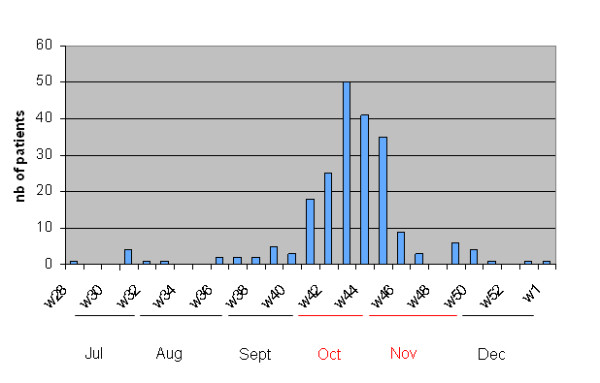
**Epidemic curve of children hospitalized with influenza A/H1N1v infection**. Brussels, July 2009-January 2010.

During the epidemic period, an excess of 18% (+10486) of pediatric outpatients and emergency department visits was registered, as compared with the mean measured over the 3 previous years during the same months. Figure 1 represents the evolution of H1N1v 2009 pediatric hospitalized cases over time in the four hospitals, with peak of the epidemic observed between the end of October and the beginning of November 2009. 215 children were hospitalized with proven or probable influenza A/H1N1v infection; representing 2% of the total hospitals' admissions registered during the whole study period but 6% (191/3144) of those during the four weeks of the peak of the epidemic. Additionally, the PICU occupation rate by influenza A/H1N1v infected children was 3.5% over the whole study period and reached 8% during the peak of the epidemic.

**Figure 2:**
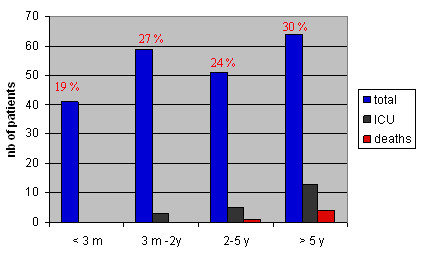
**Age distribution of the 215 hospitalized children with influenza A/H1N1v infection**. Brussels, July 2009-January 2010.

Among our cohort of 215 children, 57% were male. The median age of the patients was 31 months (range: < 1 to 208 months), with 19% of the children having less than 3 months of age (Figure 2). As shown in Table 1, 101/215 (47%) children presented with one or more underlying co-morbid condition, principally chronic lung diseases and neurological disorders. The median age of patients presenting co-morbidities was significantly higher than of those without (50 versus 14 months, p < 0.0001).

**Table 1:** Chronic co-morbid conditions in children hospitalized with influenza A/H1N1v infection in Brussels

	**Global cohort**	**Ward patients**	**PICU patients**	
	**N = 215**	**N = 194**	**N = 21**	

N of Patients with ≥ 1 co-morbidity (%)	101 (47)	88 (45)	13 (62)	p = 0.1

Distribution of co-morbidities	n (%)	n (%)	n (%)	
Chronic lung disease	32 (15)	30 (15)	2 (10)	
Asthma	18 (8)	18 (9)		
Neurological disease	31 (14)	26 (13)	5 (24)	
History of prematurity	21 (10)	21 (11)	none	
median GA* (weeks)	33 W			
Hemato-oncology	19 (9)	17 (9)	2 (10)	
HSCT**	2 (1)	2 (1)		
Sickle-cell disease	8 (4)	7 (4)	1 (5)	
Congenital heart disease	14 (7)	12 (6)	2 (10)	
surgically corrected	6 (3)			
Malnutrition	12 (6)	12 (6)	none	
Metabolic disease	3 (1)	3 (2)	none	
Diabetes	2 (1)	1 (< 1)	1 (5)	
Renal disease	3 (1)	2 (1)	1 (5)	
Solid organ transplant	2 (1)	1 (< 1)	1 (5)	

Legend:* Gestational age,** Hematopoietic cell transplantation recipients

### Clinical Presentation and Diagnosis

The major clinical features, reasons for hospitalization and blood diagnostic results are summarized in Table 2. The median duration of symptoms before admission was 2 days (IQR: 1-4 d). Presentation on admission consisted mainly in febrile respiratory illness, with a high prevalence of gastrointestinal symptoms, independently of age groups (Table 3). Moreover, 21 children (10%) presented initially with neurological manifestations. Based upon initial clinical presentation, diagnosis of influenza A/H1N1v infection was suggested by clinicians in 56% of children (Table 2). 74/215 (34%) children had chest X-ray confirmed pneumonia, associated in 6% with pleural effusion. Three patients had confirmed bacterial superinfection with *Streptococcus pneumoniae *(positive blood cultures). For five patients (2%), the influenza infection was hospital acquired.

**Table 2:** Major clinical and laboratory findings in hospitalized children with influenza A/H1N1v infection

	N = 215		N = 201¹
			
**Presentation on admission**	n (%)	**Blood test values on admission**	
H1N1 suspicion	121 (56)	Median Leukocyte count/mm3	9050
		(IQR*)	(6070-12060)
Symptoms/signs		*N (%) of patients with leukopenia ≤5000/mm^3^*	27 (13)
Fever	188 (88)	Median Lymphocyte count/mm3	2147
Cough	129 (60)	(IQR)	(1249-3632)
Rhinorrhea	105 (49)	*N (%) of patients with lymphopenia ≤1000/mm^3^*	33 (16)
Loss of appetite	58 (27)		
Vomiting	54 (25)	Median PMN** count/mm^3^	4378
Asthenia	49 (23)	(IQR)	(2667-8179)
Diarrhea	39 (18)	*N (%) of patients with neutropenia ≤1500/mm^3^*	21 (10)
Convulsion	21 (10)		
Dehydration	11 (5)	Median Platelet count/mm3	297500
Headache	11 (5)	(IQR)	(211000-381500)
Myalgia	10 (5)	*N (%) of patients with thrombocytopenia ≤150.10^3^/mm^3^*	12 (6)
Rash	7 (3)		
Abdominal pain	5 (2)	Median CRP*** (mg/dL)	1.55
Thoracic pain	5 (2)	(IQR)	(< 0.5-5.15)
		range	< 0.5-44.7
		*N (%) of patients with CRP > 5 mg/dl*	51 (25)
Reason for hospitalization	n (%)	Respiratory complications	n (%)
Altered general status	89 (41)	Hypoxemia^2^	58 (27)
Respiratory distress	78 (36)	*median oxygen saturation^3^*	90% (85-92 IQR)
IV treatment	67 (31)	*median duration of oxygen supplementation*	2.5 days
Underlying disease	53 (25)	Lobar pneumonia	57 (27)
O2 requirement	52 (24)	Diffuse pneumonia	18 (8)
< 3 months old	41 (19)	Pleural effusion	13 (6)
Seizure/malaise	22 (10)	Apnea	3 (1)
Anorexia/vomiting	14 (7)	Pneumothorax	2 (1)
« Sepsis-like » illness	6 (3)		
Parental request/non compliance	2 (1)		

Legend: *IQR = interquartile range; **PMN = polymorphonuclear leukocytes; ***CRP = C-reactive protein; 1 201 children with blood results available on admission; 2 defined as Hb oxygen saturation less than 94%; 3 transcutaneous saturation

**Table 3:** Clinical features of influenza A/H1N1v infection according to age, co-morbidity and PICU admission among hospitalized children

	Clinical presentation							
	**Pneumonia**		**Hypoxemia**		**Seizures**		GI* manifestations	
	n (%)		n (%)		n (%)		n (%)	
Global cohort (N = 215)	74 (34)		58 (27)		21 (10)		78 (36)	

Age groups (months)								
< 3 months (n = 41)	7 (17)	**p = 0.03**	4 (10)	**p = 0.01**	1 (2)	p = 0.19	13 (32)	p = 0.74
3-24 months (n = 59)	23 (39)		15 (25)		6 (10)		21 (36)	
> 24 months (n = 115)	44 (38)		39 (34)		14 (12)		44 (38)	
Co-morbidity								
yes (n = 101)	32 (32)	p = 0.4	34 (34)	**p = 0.03**	10 (10)	p = 0.95	33 (33)	p = 0.3
no (n = 114)	42 (37)		24 (21)		11 (10)		45 (39)	
PICU admission								
yes (n = 21)	15 (71)	**p < 0.001**	18 (86)	**p < 0.001**	0 (0)	p = 0.2	5 (24)	p = 0.2
no (n = 194)	59 (30)		40 (21)		21 (11)		73 (38)	

Legend:*GI = gastrointestinal manifestations, including nausea, vomiting and/or diarrhea

As shown in Table 3, children aged less than 3 months old had a significantly lower rate of pneumonia and tended to have less neurological manifestations than older ones. For this age group, the main reason for hospitalization was surveillance of acute fever without focus. Initial clinical presentation was globally similar between patients with and without chronic co-morbidities, except for hypoxemia (Table 3).

Blood inflammatory profile on admission was variable, with 13% of the children presenting with leukopenia and 6% with thrombocytopenia (Table 2). Respiratory samples collected to diagnose influenza A/H1N1v infection were nasopharyngeal aspirates and nasopharyngeal swabs in 58% and 39% of the patients, respectively. Compared to PCR (considered as the gold standard), the sensitivity of antigen by RAT was low, being only 29% (10/34) and 57% (47/83) using Coris RespiStrip and Binax Now testing, respectively (data not calculated for DIF method, as only performed on 11 samples). The sensitivity of culture was quite better at 76% (107/141).

### Treatments

Among the 215 children, only 51 (24%) received oseltamivir (doses according to weight and age [21]). For a large majority of them (37/47, data unavailable for three children), antiviral therapy was started directly on admission and was continued for five days (35/41, data unavailable for 9 patients). Rate of oseltamivir prescription reached 42% and 71% for children having pneumonia and for those requiring PICU admission, respectively. No significant related adverse events were reported. Oseltamivir was significantly more frequently prescribed among children older than 2 years compared to younger ones (Table 4) and among children with underlying disease (39/101 [39%]) versus 12/114 [11%], p < 0.0001). Additionally, 57% (123/215) of the patients were treated with antibiotics, for a median duration of seven days. In only 17 of them (14%), antibiotic therapy was discontinued after obtaining confirmation of influenza A/H1N1v infection (data unavailable for seven cases). Among children treated by antibiotics, 54% had a diagnosis of pneumonia. Finally, antibiotics were similarly used in all patients' age groups (Table 4).

**Table 4:** Co-morbidity, treatment and outcome according to age in children hospitalized with influenza A/H1N1v infection

	Children's age groups		
	< 2 years	≥ 2 years	
	N = 100 (%)	N = 115 (%)	
Rate of co-morbidity	31 (31)	70 (61)	**p < 0.0001**
Treatment			
Oseltamivir	11 (11)	40 (35)	**p = 0.001**
Antibiotics	58 (58)	65 (56)	
PICU admissions	3 (3)	18 (16)	**p = 0.002**
Invasive ventilation	2 (2)	6 (5)	
Median duration of hospitalization (d)	3	4	p = 0.13
Death	0 (0)	5 (4)	p = 0.06

### Intensive Care

21/215 patients (10%) had to be admitted to PICU, mainly within 24 hours of admission. Among them, the prevalence of co-morbidity (62%) tended to be higher than observed among ward patients, with a predominance of neurological disorders (Table 1). The median age of PICU children was 75 months (IQR 47-130). PICU admissions were significantly more frequent in children above two years of age (Table 4) and no infant less than three months old required intensive care.

The major reason for PICU admission was respiratory failure subsequent to pneumonia (Table 3). Seven patients (3% of the global cohort) presented an Acute Respiratory Distress Syndrome (ARDS) and three had pleural effusion. Two patients were admitted for surveillance because of severe underlying disease. None of the 21 children presented seizures or signs of viral encephalitis (Table 3). The median duration of PICU stay was four days and ranged from 1 to 90 days. 13/21 (59%) children received respiratory support with non invasive ventilation (NIV); eight (38%) required mechanical invasive ventilation for a median duration of six days (range 1 to 45). One previously healthy child presenting with severe ARDS followed by cardiac-respiratory arrest had to undergo Extra Corporeal Membrane Oxygenation (ECMO) support during 12 days, but survived with mild respiratory sequelae.

### Clinical Outcome

The median duration of hospitalization was three days (IQR: 2-6 d). This result was unaffected by the patients' age (Table 4).

The case-fatality rate among the global cohort was 2% (5/215). The five deaths were directly attributable to influenza A/H1N1v with or without bacterial superinfection and occurred in children with co-morbidities who would otherwise have died from their underlying disease. These underlying diseases consisted in neurological disorders from various etiologies (extensive central nervous system glioma, polymalformative syndrome, Hurler syndrome, cerebral palsy and severe encephalopathy with pontocerebellar hypoplasy). For all of them, severe pneumonia was notified, associated for three children with ARDS. All deaths concerned children aged more than two years old (Table 4). Four of the five children had received oseltamivir within 48 hours of clinical symptoms.

Three additional patients (1%) were cured from influenza A/H1N1v infection but still suffered from sequelae at the end of the study (two had bronchiectasis with emphysema and one a pulmonary restrictive syndrome needing tracheotomy and NIV support at home).

## Discussion

Even though consequences were less dramatic than initially feared, the 2009 influenza A/H1N1v pandemic has caused a significant burden of disease worldwide, especially in the pediatric population [6-8]. Higher attack rate was observed among children, causing important overload in outpatient and emergency departments [11] as well as in PICU [22]. Unfortunately, our study was not designed to assess the epidemiological impact of influenza A/H1N1v infection over the whole pediatric population of Brussels. Moreover, by selecting only laboratory confirmed infections, we underestimated the number of hospitalized cases, especially since diagnosis confirmation by PCR was no more routinely performed at the end of the epidemic. However, we were able to notice an important pediatric burden of disease in Brussels, as illustrated by an increased rate of outpatients visits of 18% during the epidemic period compared to the three previous years and a high rate of PICU occupation by influenza A/H1N1v infected patients (8% during the peak of epidemic). It would have been of interest to compare the rate of hospitalization related to H1N1 with those registered for seasonal flu during the 3 previous years but these data were unfortunately not available.

This study offers a comprehensive description of influenza A/H1N1v pattern of infection among Belgian hospitalized children, in the light of recent publications from other continents. Consistently with these reports [9-12,23,24], co-morbidities were highly prevalent among influenza A/H1N1v infected hospitalized children (47%). The co-morbidities were not different from those observed during seasonal flu. As described by others [7,9,22], the presence of at least one co-morbidity was significantly more frequent in children of more than two years of age and constituted a risk factor for severity of disease, in terms of PICU admissions and case-fatality rate. Furthermore, influenza A/H1N1v illness course differed according to patients' age groups. Indeed, children less than two years of age (46% of the cohort), and especially those less than three months, presented milder patterns of infection and were often hospitalized only for observation of fever without focus. 86% of PICU admissions and all deaths occurred in children over two years of age (with 80% of deaths among children > five years old). Although this issue is conflicting in the medical literature [7,10], similar findings have been reported in a large series by investigators from the CDC [25]. This observation differs from what is seen during seasonal flu, where young children and especially infants presented a higher mortality-rate compared to older ones [26,27]. Nevertheless, during this pandemic wave as well as during previous flu seasons, the highest rate of hospitalization was generally reported among young age groups [8,9,11,24]. This was particularly true in our series, as reflected by a median of age of 31 months, which was even younger than among American and Israeli hospitalized children (median age ranging from four to six years) [9,10]. If unexplained by the severity of infection, this finding probably illustrates differences in clinical practices and hospitalization policies. In Belgium, the National Healthcare System renders the access to inpatients pediatric facilities easy, so that hospitalization of infants presenting with fever without focus, especially those younger than 3 months of age, is largely recommended and not restricted to the most severe cases as in other countries [28,29].

Although we focused on hospitalized cases, influenza A/H1N1v episodes were mainly self-limited, consisting of febrile respiratory illness and requiring short duration of hospitalization (median 3 days) with or without oxygen supplementation. Initial clinical features did not differ from seasonal flu [30], except for the higher proportion of children presenting with gastrointestinal manifestations, as described in previous studies [12,23,24]. This involvement of the gastrointestinal tract could be subsequent to a high rate of influenza A/H1N1v virus replication [31]. Neurological manifestations were also frequent (10% of children) but in contrast with other reports [9,22] were not correlated with PICU admission or fatality. Finally, more than one third of the whole cohort and 71% of PICU patients had pneumonia confirmed on chest X-rays. Even though only 3 (1%) children had evidence of bacteremia (all due to *S. pneumoniae*), it seems very likely that a significant proportion of pneumonia, especially those with lobar infiltrates, were associated with bacterial super-infections. According to some series, bacterial super-infections after influenza A/H1N1v episodes were found in about 4% of hospitalized children [11,12,23] but reached 20 to 38% among fatal cases [7,25,32]. Considering the low rate of positive blood cultures (BC) in pediatric bacterial community-acquired pneumonia (2.5 to 5%) [33,34], these published rates as well as our data likely constitute an under-estimation, as bacterial pneumonia diagnosis relied on positive cultures from sterile sites or autopsies and as part of children had received antibiotics prior to microbiological documentation. Finally, among our whole cohort, no necrotizing pneumonia, empyema or sepsis due to *S. aureus *or group A *Streptococcus *were reported, even though those pathogens are frequently involved in other series [9,25].

Surprisingly, a majority of children were treated by antibiotics, even after the diagnosis of influenza A/H1N1v infection was obtained. As mentioned above, confirming bacterial pulmonary super-infection after influenza illness is challenging and diagnostic relies more on clinical presumption and unspecific blood results [35]. However, this could only partly explain the high rate of antibiotics use, as only 54% of those children treated by antibiotics presented pulmonary infiltrates. Rate of antibiotics prescriptions was uniform among all age groups and was also high in other pediatric studies [10-12]. The exact reasons sustaining this practice remain unknown but should be worth to investigate in further prospective studies. Contrastingly, our study showed a particularly low percentage of oseltamivir prescriptions. Indeed, only 24% of the children were treated compared with 45 to 84% in other similar studies [8,9,11,12]. In our four centers, the use of oseltamivir was significantly higher in children above 2 years of age and/or suffering from co-morbidities. This more "watch and wait" practice was in line with the restrictive national recommendations issued for oseltamivir pediatric use during the 2009 pandemic wave, which suggested cautious prescription under one year of age, regarding the absence of safety data among infants [16]. Moreover, in Belgium, prescription of antiviral drugs during seasonal flu is very limited and kept for management of severe diseases or immuno-compromised patients [36]. Obviously, the limitations associated with the retrospective design do not allow us to conclude on treatment efficacy. It is however interesting to note that, although oseltamivir was scarcely used, fatality rate and PICU admissions were comparable to the other above-mentioned reports [9,10,12].

In Argentina [8], the case-fatality rate of influenza A/H1N1v infected children was 5%, with a global pediatric mortality rate 10 times greater compared to previous flu seasons. National surveys in the United Kingdom [7] and U.S [23] reported also a higher influenza related mortality rate during the pandemic influenza A/H1N1v than observed with seasonal flu. However, consequences in the Northern hemisphere were less dramatic than anticipated. Studies from these countries reported case-fatality rate among hospitalized children ranging from 0.6 to 3% [9-12], similar to our findings (2%). As previously hypothesized [10], these North/South differences in patients' outcome could partly be explained by an easier access to the health care system in Europe, Israel and North America. In Israel [10], as well as in our series, the median duration of symptoms before hospitalization was only 2 days compared to 4 days in Argentina [8]. On another hand, 2 patterns of influenza A/H1N1v related deaths have been described [7]: those occurring after several days of hospitalization in chronically ill patients (80%), in contrast to those observed after acute evolution of viral infection in previously healthy children (20%). Despite a small number of cases, a similar profile seemed to happen in our series, as the five children who died suffered from chronic neurological disorders and one previously healthy child presented a fulminant viral infection causing cardio-respiratory arrest and requiring nine days of ECMO support to be cured.

## Conclusion

Although influenza A/H1N1v infections were globally self-limited, pediatric burden of disease was significant. Children of more than 2 years old and/or suffering from chronic co-morbidities were shown at higher risk of severe infection. Compared to other countries experiencing different health care systems, our Belgian cohort was younger and received less frequently antiviral therapy; disease course and mortality were however similar.

## Competing interests

The authors declare that they have no competing interests.

## Authors' contributions

SB, EH, IDS, JL, DM, AV, AM and PL designed the study. SB, EH, MCC, CF, IDS, RA, DH, MCN, BM, CF and GVB collected patients' data. MR, IW and BKM performed microbiological testing. SB and PL performed data analysis. SB, EH and PL drafted the report. MCC, CF, IDS, RA, DH, MCN, BM, CF, GVB, JL, DM, AV and AM revised the report. All authors read and approved the final manuscript.

## Pre-publication history

The pre-publication history for this paper can be accessed here:

http://www.biomedcentral.com/1471-2334/11/313/prepub
